# The effects of a multi-ingredient supplement on markers of muscle damage and inflammation following downhill running in females

**DOI:** 10.1186/s12970-016-0156-5

**Published:** 2016-11-25

**Authors:** Jessica L. Köhne, Michael J. Ormsbee, Andrew J. McKune

**Affiliations:** 1 Discipline of Biokinetics, Exercise and Leisure Sciences, School of Health Sciences, University of KwaZulu-Natal, Durban, South Africa; 2 Department of Nutrition, Food and Exercise Sciences, Institute of Sport Sciences and Medicine, Florida State University, Tallahassee, USA; 3 Discipline of Sport and Exercise Science, University of Canberra Research Institute for Sport and Exercise, Faculty of Health, University of Canberra, Canberra, Australia

**Keywords:** Eccentric exercise, Muscle soreness, Recovery

## Abstract

**Background:**

The effects of a multi-ingredient performance supplement (MIPS) on markers of inflammation and muscle damage, perceived soreness and lower limb performance are unknown in endurance-trained female athletes. The purpose of this study was to determine the impact of MIPS (NO-Shotgun®) pre-loaded 4 weeks prior to a single-bout of downhill running (DHR) on hsC-Reactive Protein (hsCRP), interleukin (IL)-6, creatine kinase (CK), muscle soreness, lower limb circumferences and performance.

**Method:**

Trained female runners (*n* = 8; 29 ± 5.9 years) (VO_2max_: ≥ 50 ml^-1^.kg^-1^.min^-1^, midfollicular phase (7-11 days post-menses) were randomly assigned in a double-blind manner into two groups: MIPS (*n* = 4) ingested one serving of NO Shotgun daily for 28 days prior to DHR and 30 min prior to all post-testing visits; Control (CON) (*n* = 4) consumed an isocaloric maltodextrin placebo in an identical manner to MIPS. hsCRP, IL-6, CK, perceived soreness, limb circumferences, and performance measures (flexibility, squat jump peak power) were tested on 5 occasions; immediately before (PRE), immediately post-DHR, 24, 48 and 72 h post-DHR.

**Results:**

There were main effects of time for CK (*p* = 0.05), pain pressure threshold (right tibialis anterior (*p* = 0.010), right biceps femoris (*p* = 0.01), and left iliotibial band (ITB) (*p* = 0.05) across all time points), and maximum squat jump power (*p* = 0.04). Compared with 24 h post-DHR, maximum squat jump power was significantly lower at 48 h post-DHR (*p* = 0.05). Lower body perceived soreness was significantly increased at 24 h (*p* = 0.02) and baseline to 48 h (*p* = 0.02) post DHR. IL-6 peaked immediately post-DHR (*p* = 0.03) and hsCRP peaked at 24 h post-DHR (*p* = 0.06). Calculation of effect sizes indicated a moderate attenuation of hsCRP in MIPS at 72 h post-DHR.

**Conclusions:**

Consumption of MIPS for 4 weeks prior to a single bout of DHR attenuated inflammation three days post, but did not affect perceived soreness and muscle damage markers in endurance trained female runners following a single bout of DHR.

## Background

Exhaustive or unaccustomed high-intensity exercise [1, 2] or eccentric contractions [3, 4] are known to inflict muscle damage. This leads to increased muscle soreness, decreased muscle strength, increased concentration of plasma muscle enzymes, and decreased range of motion [1, 2, 4], for several days post exercise, and further promotes an unbalanced ratio of protein breakdown to protein synthesis [5]. A level of muscle discomfort related to the exercise-induced muscle damage (EIMD), otherwise known as delayed onset muscle soreness (DOMS), increases until subsiding five to seven days post-exercise [6]. Downhill running (DHR) is commonly used to induce muscle damage [7], as it involves greater eccentric contraction of the knee extensors, leading to more tissue damage, compared with level or uphill running [8, 9].

Although similar decrements associated with EIMD have been reported in both males and females, it has been documented that females are susceptible to longer periods of recovery when compared to males; however, reasons for this are unclear [10]. However, contrary to this, estrogen appears to have a protective effect with regards to DOMS [11], as it has been reported that estrogen results in significant decreases in serum creatine kinase (CK) activities post-exercise or post-injury when compare to men or a placebo [12, 13]. It has therefore been hypothesized that several of the sex-related differences are attributed to the female sex hormone 17β-estradiol [14, 15]; such that estrogen has been reported to possess the ability to enhance skeletal muscle growth, gene expression, metabolism, contraction characteristics, and maintain muscle mass.

Numerous studies have examined the effects of nutritional supplementation interventions to reduce the effects of EIMD by increasing the net protein balance within skeletal muscle. These studies have included supplementation with whey protein isolate [5, 16], branched-chain amino acids (BCAAs: leucine, isoleucine, and valine) [17, 18], protein hydrolysates [19], leucine only [20], creatine [21]; increasing anti-inflammatory and anti-oxidant activity through tart cherry juice [22, 23], blueberry [24] and caffeine ingestion [25], as well as enhancing recovery through the consumption of chocolate milk [26, 27] to name a few. The majority of studies investigating the ergogenic effects of nutritional supplementation on exercise performance and recovery have primarily focused on males [20, 28, 29]. However, there are a few studies that examined female rats and other animals, and female humans [30, 31].

Recent studies have investigated the effects of a multi-ingredient performance supplement (MIPS; NO Shotgun®) following a single bout of DHR in trained males [32] as well as in resistance trained males [33]. However, there is little known about the effects of this MIPS on the reduction of muscle damage and repair after EIMD from DHR in endurance-trained female athletes. We hypothesized that 4 weeks of pre-loading with a MIPS prior to a single bout of DHR would decrease perceived soreness and circumferences, enhance flexibility, and improve biochemical markers of muscle damage and inflammation (i.e. CK, IL-6 and CRP), and improve squat jump performance when compared to an isocaloric placebo (CON) in endurance-trained female runners for up to 72 h post-DHR.

## Methods

All procedures were carried out in the Human Performance Laboratory situated at the University of KwaZulu-Natal (UKZN) Discipline of Biokinetics, Exercise and Leisure Sciences following approval from the UKZN Biomedical Research Ethics Committee (Ethics Approval Number: BFC033/13).

### Participants

Eight healthy female endurance-trained runners (maximal oxygen uptake (VO_2max_) ≥ 50 mL^-1^.kg^-1^.min^-1^, average 32 km.wk^-1^ of running), aged 18-40 years, across all ethnic groups from Durban, KwaZulu Natal, South Africa and the surrounding areas were recruited to participate in this study (Table 1). Participants were excluded if they had any existing diseases and/or musculoskeletal disorders, history of leg injury or any other medical condition that would be exacerbated by a single bout of DHR, or the regular use of any anti-inflammatory drugs, and suffering from allergies. Participants were requested to discontinue the use of any other dietary or ergogenic supplementation and were asked to complete a washout period of four weeks prior to participation in the study [32, 34] with the exception of a multivitamin without known ergogenic enhancers (one participant reported using a multi-vitamin, however, no other supplement use was reported). Participants did not take any supplement other than the provided MIPS or CON, for the duration of the study. Throughout the study all participants maintained their habitual diet, and were given 24 h dietary food logs to record their meals the day prior to baseline testing and were asked to replicate these meals before all other laboratory visits. Participants were required to document their dietary intake for two week days and one weekend day for the four weeks of supplementation prior to completing the DHR. Automated Self-administrated 24-h recall (ASA24) online dietary software (http://asa24.westat.com) was used to analyze the participants’ diets, to ensure that their diets had not changed during the testing period. Participants ate their last meal at least 6 h prior to the DHR, and consumed a commercial PVM Energy Bar® (183.40 kcal) 3 h prior to testing.

**Table 1:** Participant Characteristics (*N* = 8)

	MIPS (*n* = 4)	CON (*n* = 4)	TOTAL (*n* = 8)
Age (yrs)	28.5 (±6.5)	29.5 (±5.123)	29 (±5.874)
Height (m)	1.66 (±0.06)	1.60 (±0.06)	1.63 (±0.06)
Body Mass (kg)	57.06 (±3.00)	56.40 (±4.90)	56.73 (±3.78)
BMI (kg.m^-2^)	20.78 (±0.68)	22.03 (±1.69)	21.40 (±1.37)
VO_2_max (mL.kg^-1^.min^-1^)	56.68 (±3.12)	59.95 (±3.23)	58.31 (±2.17)
Body Fat (%)	15.31 (±1.82)	14.58 (±1.57)	14.95 (±1.62)

Values are expressed as mean (± SD)

*MIPS* Multi-Ingredient Performance Supplement, *CON* Control (Isocaloric Placebo), *VO*_*2max*_ Maximal Oxygen Consumption, *BMI* Body Mass Index

All participants reported running more than 30 km per week, for more than three time a week, exercising at either a moderate or high intensity.

### Study design

This was a randomized, placebo-controlled, double-blind study. After baseline testing, subjects were randomly assigned to either a MIPS group (*n* = 4) or an isocaloric flavour-matched placebo control (CON) group (*n* = 4). The MIPS group was required to consume one serving (21 g) of a multi-ingredient sports supplement (NO-Shotgun®, Vital Pharmaceuticals, Inc., Davie, FL) containing ~72 kcals; 18 g protein; 9.7 g protein hydrolysate matrix including BCAAs; 8.06 g muscle volumizing and 3.17 g power/speed/strength and endurance matrix that includes multiple forms of creatine and beta alanine; 376 mg of Redline® Energy matrix including caffeine; once a day for four weeks prior to the DHR. The CON group was required to consume an isocaloric, flavour-matched placebo beverage (maltodextrin, 28 g) once a day for four weeks prior to the DHR. All participants received their supplement in identical commercially labelled containers after baseline testing. Subjects consumed either MIPS or CON 30 min prior to exercise on reported training days, or first thing in the morning on all non-training and recovery days.

### Experimental design

**Fig. 1:**
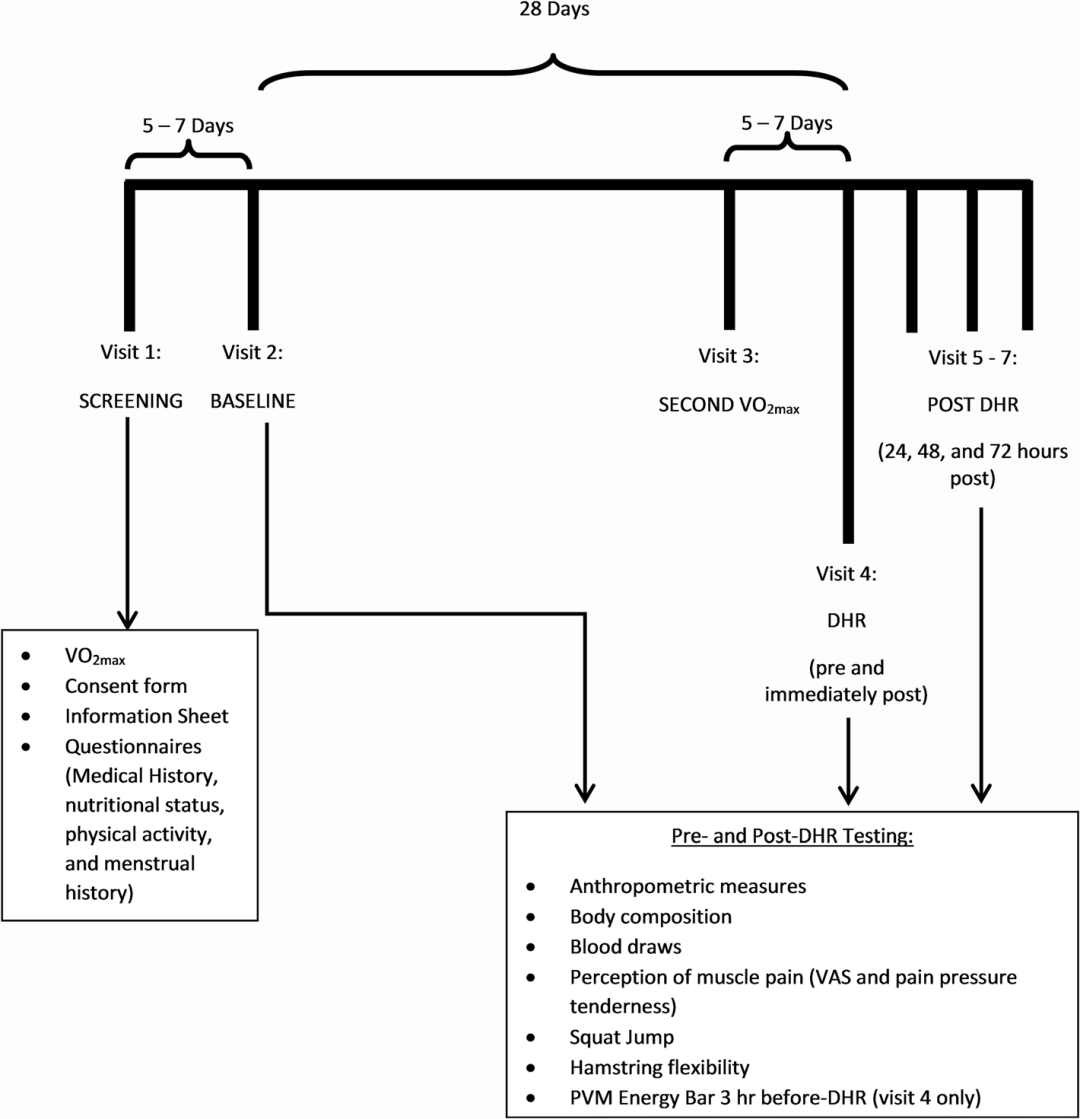
Study timeline. VAS: Visual Analog Scale; VO_2max_: Maximal Oxygen Uptake. Multi-Ingredient Performance Supplement (MIPS) or Control (CON) consumed daily from visit 2 up to 1 day prior to visit 4 (30 min prior to exercise on training days, and immediately upon waking on non-training days), as well as 30 min prior to participants arriving for Visit 5-7

Participants reported to the laboratory on 7 different occasions (Fig. 1). The first visit included initial screening for inclusion of the participants, including VO_2max_ determination, and completion of written consent to participate in the study. An incremental treadmill running protocol was employed to determine VO_2max_ using a motor driven treadmill (h/p/cosmos Cosmed™ T150). Expired air was measured breath-by-breath indirect calorimetry using a metabolic measurement system (Cortex MetaLyzer® 3B, Cortex Biophysik, GmbH). Prior to the beginning of each test, the gas analyzer was calibrated using ambient air and a gas of a known composition containing 20.9% oxygen (O_2_) and 4% carbon dioxide (CO_2_). The turbine flowmeter was calibrated using a 3-L syringe. Heart rate (HR) was monitored using a Polar™ Heart Rate Monitor (Accurex 2, Finland). Ratings of perceived exertion (RPE) and HR were measured every minute. The initial velocity and inclination were set at 8.0 km.hr^-1^ and 3%, respectively, for 5 min as a warm-up. The velocity and inclination then increased in increments of 1 km.hr^-1^ and 1% every minute until a speed of 12 km.hr^-1^ was reached, with the incline continuing to increase until exhaustion. The test was considered to be a maximal test under two conditions: one being if the participants oxygen uptake plateaued for more than two stages (2 min intervals) while intensity continued to increase, and the other being that participants exhibit two of the following secondary criteria: RPE ≥ 19, respiratory exchange ratio (RER) ≥ 1.1, or a heart rate within 10 beats∙min^-1^ of the theoretical age-predicted maximum HR (220-age) [35]. The second visit was no later than one week from the initial visit and was used for baseline testing. Baseline testing included all measures indicated below. Upon completion of baseline testing the participants were given instructions on supplementation and instructed to maintain their normal dietary and training patterns for the next 28 days during supplementation. Participants were required to complete questionnaires regarding nutritional status and a medical history, physical activity, and menstrual history. While training logs were not kept, verbal verification was obtained to confirm that participants did not alter their training schedule. Approximately 3 weeks from baseline testing participants returned to the laboratory under the same pretesting conditions as baseline to perform a VO_2max_ test to determine their intensity (75% of VO_2max_) during the DHR. This visit was further used to familiarize the participants to the treadmill. The visits were all scheduled during the morning hours (0500 h – 0800 h) in order to control the variables being tested.

A 15 mL venous blood sample was collected and perceived muscle soreness, pain pressure threshold/tissue tenderness, squat jump performance, circumferences, and flexibility were measured, at the 5 different laboratory visits: baseline (28 days before DHR), immediately before DHR, Immediately post DHR, and 24, 48 and 72 h post-DHR. Following the blood draws, muscle soreness and tissue tenderness measurements were taken, participants were required to complete a warm-up walk for a duration of five minutes at 4.8 km.hr^-1^ on a motorized treadmill, prior to any performance measurements. The DHR was performed on visit 4 (Fig. 1). Prior to completing the DHR, participants consumed their commercial PVM Energy Bar® 3 h before testing, and replicated their 24 h dietary food logs.

### Measures

Height and body mass were measured using a Sanny stadiometer and a balance scale (Detecto Medic, Detecto Scales Inc., Brooklyn, N.Y., U.S.A.) respectively. All measurements were taken without shoes and any excess clothing and accessories. Body composition was measured non-invasively using the sum of 7 skinfold measurements (triceps, chest, mid-axillary, subscapular, supra-iliac, abdominal, and thigh), all taken by the same technician, that were used to calculate body fat percentage [36].

Blood (15 mL) was collected via venipuncture of the antecubital vein at pre, immediately post-, 24, 48 and 72 h post-DHR. Following blood collection, serum was allowed to clot before centrifuging at 3500 RPM at 4 °C for 15 min (Sorvall ST16R Multispeed Centrifuge; Thermo Electron Corporation, Needham Heights, Massachusetts). Aliquots of 300 μL each were then transferred into microtubes and immediately frozen at -80 °C for later batch analysis. Blood samples were sent to a commercial pathology laboratory for analysis of serum markers of muscle damage and inflammation (CK, IL-6, hsCRP). CK and hsCRP were analyzed using Roche/Hitachi cobas c 311, cobas c 501/502 analyzers (Roche Diagnostics GmbH). IL-6 was analyzed using the Access IL-6 assay (Beckman Coulter, Inc.).

Circumferences of the lower limbs were measured to determine the volume changes in the thigh as an indirect marker of edema. A standard centimetre marked tape measure was utilized to measure changes in muscle girth. The participants were measured at the followings areas: 10 and 15 cm above the superior pole of the patellae, on the quadriceps muscle, as well as along the midline of the calf muscle 15 cm from the joint margin of the knee, and hamstring circumference was measured at the midline from the ischial tuberosity to the lateral epicondyle of the tibia. Perceived muscle soreness of the lower body was measured using a Visual Analogue Scale (VAS) [37]. Participants were requested to rate the level of soreness experienced in the anterior thigh of their left leg by drawing an “X” across a continuum line extending from 0 cm (no soreness) to 10 cm (extreme soreness). To quantify the pressure pain threshold or tissue tenderness of the lower limbs and gluteal muscles, an algometer (Wegner Force Ten ™, Wegner Instruments, Greenwich, CT) was used with an application of at 1 kg.cm^-2^.sec^-1^ of pressure [38]. The footplate of the algometer was positioned perpendicular to the muscle belly.

Hamstring flexibility was measured using a sit-and-reach box using standard testing procedures [36]. Each participant performed three trials of the sit-and-reach test, of which the best value was used for analysis [39].

Dynamic explosive force production of the leg extensors was assessed during a squat jump test recorded using the MyoTest PRO2 (MyoTest performance measurement system) [40] to determine the height, velocity, and power of the jump. Participants were instructed to assume a squat position, with their knees shoulder width apart, to a knee angle of 90° and explosively jump as high as possible. Participants completed three jumps with a 30 s break between each jump, such that the mean was recorded for analysis.

### Downhill running protocol

Participants were tested during the mid-follicular phase (7-11 days post-menses) of the menstrual cycle. None of the participants reported using any form of oral contraceptive. Prior to the DHR on visit 4, participants completed a warm-up run at 8 km.hr^-1^ for five minutes on a level gradient. Participants were required to perform a jump squat before the warm-up. Once the warm-up was completed, the treadmill gradient was lowered to -10%. The participants continued running for a total duration of 60 min at a previously-determined velocity that generated 75% of their VO_2max_ on a level grade (determined during visit 3; using the ACSM metabolic equation [41]). Heart rate (HR) and rating of perceived exertion (RPE, Borg CR10 scale) [42] were monitored continuously and recorded every five minutes during the DHR protocol. Following 30 min of DHR, participants were required to perform a squat jump, if a 40% force decrement was evident compared to the jump-squat measures for prior to the DHR [43], the participant did not have to continue running, however if no decrement was evident, the participant continued running for the full 60 min.

### Statistical analyses

Normal distribution of the variables was checked by the Shapiro-Wilk Test. In addition, visual inspection of the data distribution was performed. The test indicated that creatine kinase (CK) was not normally distributed (*p* < 0.01) and therefore a natural logarithm (Ln) was applied. After Ln-transformation, CK was normally distributed. Once CK was transformed, the generalized linear model (GLM) was used on all the data for each parameter tested to identify any significant changes within each subjects set of data, between subjects and over time using a factorial repeated measures ANOVA (2x5). Where appropriate pair wise multiple comparisons were performed using the Bonferroni post-hoc test. *p* ≤ 0.05 was considered statistically significant. Cohen’s *d* effect sizes (ES) and 95% confidence intervals (CI) were also calculated for all out come measures. Magnitudes of the standardized effects were interpreted using thresholds of <0.2, 0.2-0.6, 0.6-1.2, 1.2-2.0, 2.0-4.0. These values correspond to trivial, small, moderate, large and very large ES, respectively. However, effects were only claimed as clear if the confidence interval did not cross both the positive and negative thresholds. Statistical analyses were performed using the IBM SPSS Statistics for Windows, Version 22.0. (Armonk, NY: IBM Corp).

## Results

### Downhill run protocol

The speed used for the DHR was 12.03 (±0.4) km.hr^-1^ for both groups combined, with the control having a higher speed compared to the supplement group (12.28 (±0.8) km.hr^-1^ vs. 11.78 (±0.2) km.hr^-1^). During the DHR, the average HR for both groups combined was 139.72 (±4.5) b.min^-1^, Table 2 indicates the average heart rates for both the MIPS and CON groups during the DHR protocol. Furthermore, the RPE values remained relatively low throughout the DHR, with similar values being experienced in both groups (Table 2). No significant treatment effects were noted for the treadmill speed (*p* = 0.6), HR (*p* = 0.5), and RPE (*p* = 0.8). None of the participants experienced a 40% decrement in their squat jump force at 30 min into the DHR, and as such all participants ran for the full 60 min. With regards to the participant’s diets, the amount of protein (72.51 ± 27.62 g vs 88.00 ± 8.79 g), fats (85.43 ± 35.29 g vs 86.12 ± 39.65 g) and carbohydrates (165.45 ± 54.48 g vs 177.11 ± 32.93 g) did not vary substantially between the supplement and placebo groups respectively (*p* > 0.05). Overall energy intake did not vary significantly between the supplement and placebo groups (1690.80 ± 213.31 kCal vs 1846.09 522.20 kCal; *p* = 0.355).

**Table 2:** Average heart rate (HR) and ratings of perceived exertion (RPE) during the DHR protocol

GROUP	HR (b.min^-1^)	RPE
MIPS	143.08 (±7.0)	3.64 (±0.6)
CON	136.36 (±6.4)	3.32 (±0.7)
Total	139.72 (±4.5)	3.48 (±0.4)

Values are expressed as mean (± SD); no significant differences were notes (*p* ≤ 0.05)

### Response to EIMD protocol

#### Circumferences

No significant time or time x treatment effects were evident for all lower limb circumferences (Quadriceps, Hamstrings, Calf and Gluteus Maximus). With no significant difference between left and right quadriceps (*p* = 0.59), hamstrings (*p* = 0.52), and calves (*p* = 0.94) over time for combined data. Effect sizes as demonstrated by Cohen’s *d* <0.2 were trivial for both groups (data not shown).

#### Perceived muscle soreness

**Fig. 2:**
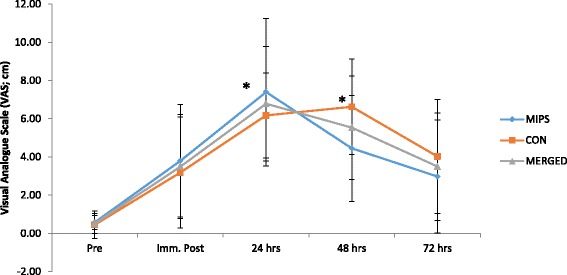
Perceived muscle pain values (VAS), prior to and following the DHR protocol. Pre, immediately post, 24, 48 and 72 h post the DHR. Main time effect for merged data with a significant difference between baseline and 24 h post (*p* = 0.02), and baseline and 48 h post (*p* = 0.02) (* denotes significance *p* ≤ 0.05)

Mean VAS measures before and after the DHR are presented in Fig. 2. There was no group x time interaction; however there was a time effect with VAS following the DHR, peaking at 24 h post. Significant differences were found between pre- and 24 h post-DHR (*p* = 0.02), and pre- and 48 h post-DHR (*p* = 0.02). The VAS measurements at 24 h post-DHR increased 6-fold from baseline values, whilst those at 48 h exhibited a 5-fold increase from baseline. Effect sizes for both groups were similar at all time-points post downhill run ranging from large to very large increases in perceived muscle soreness (Table 3).

**Table 3:** Effect sizes and interpretation for VAS, Peak Power, CK, IL-6 and CRP. Pre versus immediately post (IP), 24, 48 and 72 h post DHR

Outcome	Time	MIPS	Int	CON	Int
VAS	Pre vs IP	1.8 (0.5, 3.0)	Large	1.5 (0.2, 2.8)	Large
	Pre vs 24 h	2.9 (1.2, 4.5)	Very large	4.0 (3.0, 5.0)	Very large
	Pre vs 48 h	2.3 (1.1, 3.4)	Very large	3.9 (2.8, 5.0)	Very large
	Pre vs 72 h	1.3 (0.1, 2.6)	Large	1.9 (0.6, 3.2)	Large
Peak Power	Pre vs IP	0.2 (-3.3, 3.6)	Unclear	0.2 (-5.1, 5.5)	Unclear
	Pre vs 24 h	0.3 (-2.9, 3.5)	Unclear	0.3 (-4.1, 4.6)	Unclear
	Pre vs 48 h	1.2 (-1.9, 4.2)	Unclear	0.8 (-3.6, 5.3)	Unclear
	Pre vs 72 h	0.9 (-2.5, 4.2)	Unclear	0.4 (-5.2, 5.9)	Unclear
CK	Pre vs IP	1.3 (-1.4, 1.2)	Unclear	0.5 (0.2, 0.9)	Small
	Pre vs 24 h	1.4 (1.1, 1.7)	Large	1.9 (1.6, 2.2)	Large
	Pre vs 48 h	1.0 (0.8, 1.2)	Moderate	1.1 (0.8, 1.4)	Moderate
	Pre vs 72 h	1.0 (0.9, 1.1)	Moderate	0.4 (0.1, 0.7)	Small
IL-6	Pre vs IP	2.5 (2.1, 2.9)	Very large	1.1 (0.7, 1.6)	Moderate
	Pre vs 24 h	1.6 (1.4, 1.9)	Large	0.2 (0.2, 0.6)	Small
	Pre vs 48 h	0.3 (0.3, 0.9)	Small	0.4 (0.4, 1.3)	Small
	Pre vs 72 h	0.4 (0.2, 1.1)	Small	0.5 (0.8, 1.7)	Small
CRP	Pre vs IP	-0.1 (-0.3, 0.1)	Unclear	-0.1 (-0.3, 0.2)	Unclear
	Pre vs 24 h	0.1 (-0.1, 0.3)	Unclear	0.2 (-0.2, 0.7)	Unclear
	Pre vs 48 h	-0.5 (-0.3, 0.6)	Unclear	1.9 (1.5, 2.3)	Large
	Pre vs 72 h	-0.9 (-1.0, -0.7)	Moderate	0.8 (0.2, 1.4)	Moderate

The pressure pain threshold or tissue tenderness was quantified using a force algometer. Significant time effects were only noted for the right tibialis anterior (*p* = 0.01), right biceps femoris (*p* = 0.01), and the left iliotibial band (ITB) (*p* = 0.05) for combined data for both groups across all time points (Fig. 4). Combined data of tissue tenderness showed that majority of the areas experienced increased tenderness (decreased algometer force), from baseline; however, the right hip flexor values remained above baseline across all time points; and the left hip flexor values increased at 24 h, decreasing at 48 h but remaining above baseline, and then decreasing further beyond baseline, at 72 h following the DHR protocol. Effect sizes as demonstrated by Cohen’s *d* <0.2 were trivial for both groups (data not shown).

#### Hamstring flexibility

No significant time x treatment (*p* = 0.81) or time effect (*p* = 0.43) was noted for hamstring flexibility for combined data. Effect sizes as demonstrated by Cohen’s *d* <0.2 were trivial for both groups (data not shown)..

#### Jump performance

**Fig. 3:**
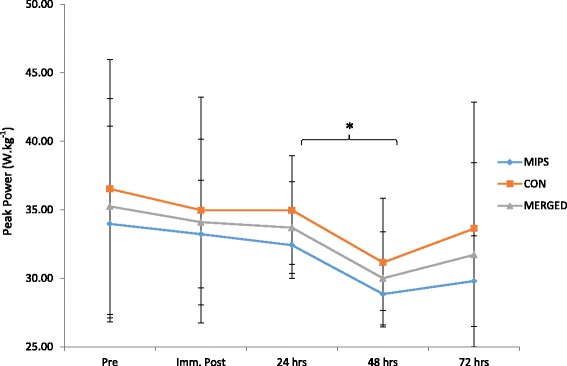
Squat jump peak power. Significant difference between 24 and 48 h following the DHR protocol for merged data (*p* = 0.05) (* denotes significance *p* ≤ 0.05)

Average power, velocity, force and jump height did not exhibit any significant group x time or time effects. However, peak power (W.kg^-1^) showed a significant time effect (*p* = 0.04), with a significant difference occurring between 24 and 48 h post-DHR (*p* = 0.05) (Fig. 3). Effect sizes for both groups were unclear post-DHR (Table 3).

#### Serum markers of muscle damage and inflammation

**Fig. 4:**
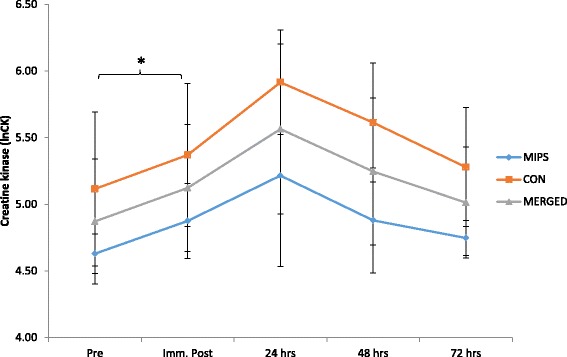
Normalized serum Creatine Kinase (CKLog) values prior to and following the DHR protocol. Main time effect for merged data with a significant difference between pre and immediately post DHR (*p* = 0.03) (* denotes significance *p* ≤ 0.05)

**Fig. 5:**
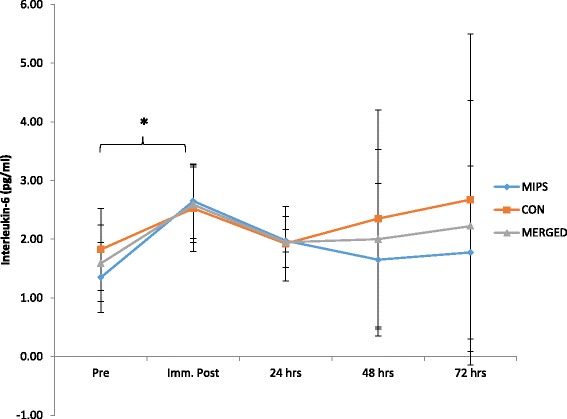
Interleukin-6 (IL-6) values before and after the DHR protocol. Main time effect for merged data with a significant difference between pre- and immediately post-DHR (*p* = 0.03) (* denotes significance *p* ≤ 0.05)

**Fig. 6:**
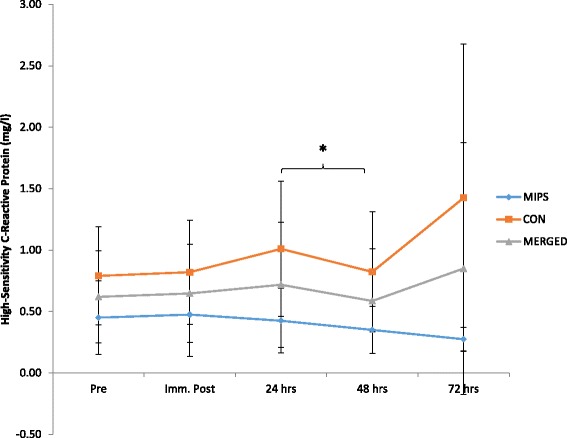
High-sensitivity C-Reactive Protein (hsCRP) values before and after the DHR protocol. Main time effect for merged data with a significant difference between 24 and 48 h post-DHR (*p* = 0.04). (* denotes significance *p* ≤ 0.05)

There was no significant time x group effects for CK_Log_ (*p* = 0.25); however, there was a time effect with a significant increase from baseline to immediately post-DHR (*p* = 0.02) (Fig. 4). IL-6 did not show a significant group x time effect (*p* = 0.80); however, there was a significant (*p* = 0.03) 62.99% increase immediately post the DHR (Fig. 5). There was no group x time effect for hsCRP (*p* = 0.55), however, there was a time effect with a significant decrease in hsCRP between 24 and 48 h post-DHR (*p* = 0.04) (Fig. 6). Effect sizes for both groups were similar post downhill run for increases in CK and IL-6 ranging from small to very large increases (Table 3). Based on ES, CRP was the only variable to demonstrate contrasting results. Effect sizes were trivial for both MIPS and CONT up to 24 h post-DHR. However, at 72 h post-DHR, MIPS demonstrated a moderate decreases whilst CON demonstrated moderate increase (Table 3).

## Discussion

The main findings of this study were that consumption of MIPS for 4 weeks prior to a single bout of DHR did not attenuate the changes of ratings of perceived soreness, muscle damage and performance (flexibility, squat jump peak power), greater than an isocaloric placebo (CON) in well-trained female endurance runners for up to 72 h post-DHR. However, based on ES, CRP, a marker of inflammation, was moderately attenuated at 72 h in MIPS compared with CON that exhibited a moderate increase.

### Delayed onset muscle soreness

Delayed onset muscle soreness (DOMS) generally develops 24 – 48 h following exercise, peaks at 24 – 72 h, and typically subsides five to seven days post-exercise [6, 32, 44]. Two days following DHR, tissue tenderness, measured by pressure transducers, has shown to be greatest in the gluteus maximus, rectus femoris, vastus medialis, vastus lateralis, tibialis anterior, gastrocnemius, and the biceps femoris [45]. Our findings confirmed that the greatest amount of perceived muscle soreness (VAS and pressure algometer measurements) was reported during 24 and 48 h following the DHR, with no difference between treatment groups. Additionally, Ormsbee et al. [32] indicated greater perceived muscle soreness following a single-bout of DHR in the rectus femoris, vastus medialis, vastus lateralis, biceps femoris and gluteus maximus of the right leg compared to prior to completing a DHR. The findings of our study are in agreement with these findings. Furthermore, the findings of this study are in accordance with findings from other DHR-induced muscle damage studies [8, 32], as well as a study on female athletes following a repeat sprint protocol, in which VAS was reported to peak at 48 h post [46].

Several studies have attempted to reduce DOMS and stimulate protein synthesis following strenuous exercise through supplementation with whey protein isolate [16]. Acute ingestion of protein (100 g protein containing 40 g EAAs) immediately following a single 30 min bout of DHR did not attenuate DOMS during 72 h of recovery [47]; however, BCAA supplementation (2.5 g BCAAs ingested immediately prior to and during exercise) has shown to significantly decrease DOMS from 24 to 72 h post-endurance exercise [48]. Matsumoto et al. [49] further support this finding of reduced DOMS with BCAA supplementation (0.8% total BCAA: 5 g valine, 10 g leucine, 5 g isoleucine; 0.2% arginine; and 3.5% CHO). Moreover, Green et al. [50] demonstrated that ingestion of a supplement containing carbohydrates (CHO) and protein one hour (1.2 g CHO.kg BW^-1^ with 0.3 g Protein.kg BW^-1^), and two hours (0.6 g CHO.kg BW^-1^ with 0.15 g Protein.kg BW^-1^) following 30 min of DHR at -12% gradient in females did not reduce the amount of DOMS experienced, when compared to ingestion of CHO only. Additionally, Green et al. [50] did note that muscle soreness peaked at 48 h post-DHR, and decreased, but remained elevated above baseline, at 72 h for both groups. In the present study, no significant difference was reported in perceived muscle soreness between groups, which is in agreement with others using protein [47, 50], BCAAs [17, 51], leucine only [20], or creatine [21, 52]. Irregularities in studies identifying the effects of supplementation on EIMD could be due to varying individual dosages of each individual supplement in previous studies compared to the MIPS used in this study.

### Jump performance and flexibility

Decrements in strength performance measures, including jump performance, following eccentric exercise have been well documented [20, 53]. According to Eston et al. [45], an acute loss of strength following DHR is the most noticeable effect of the eccentric nature of the run. Following a dance muscle damaging protocol in female dancers, Brown et al. [54] indicated that counter-movement jump (CMJ) measurements returned to near baseline levels by 24 h post damaging exercise, while Keane et al. [46] showed return to near baseline of CMJ at 72 h following a repeat sprint protocol. Supplementation with NO-Shotgun® was not effective in attenuating the strength decrements observed following the DHR. The average squat jump power, peak squat jump power, squat jump velocity, and jump height and force all indicated peak decrements 48 h post-DHR. This is in accordance with previous research, indicating that the strength decrements following DHR will require longer recovery time compared to other forms of muscle damaging protocols [31], as well as being in accordance with findings elicited in a study supplementing with NO-Shotgun® following DHR in male participants [32]. The delayed recovery in strength/power output following DHR in females was also noted by Green et al. [50]. Furthermore, the lack of effect of supplementation on recovery in strength has been observed in studies supplementing with protein [50] and creatine [52], while others have observed a positive effect of supplementation on strength decline following EIMD [5, 22, 55].

As previously indicated, EIMD is characterized by both strength and range of motion decrements. Proske, Morgan [56] noted that eccentric exercise leads to an increase in passive tension in the muscle, and indicated that muscular stiffness increased immediately post- and remained elevated up to four days post-exercise. A sit-and-reach test was used to assess the flexibility of the hamstrings following the DHR protocol. Our findings suggest that there was no significant change in hamstring flexibility following the DHR in both the MIPS and CON groups, this is in agreement with findings found by Ormsbee et al. [32]. Furthermore, it appears that although pain was experienced in the hamstrings, the flexibility of the hamstrings was not significantly compromised due to the DHR-protocol, as DHR results in greater damage to the knee extensor muscles in comparison to level running [8, 9]. Supplementation with NO-Shotgun® did not elicit significant improvements in hamstring flexibility in comparison to an isocaloric placebo.

### Biochemical markers of muscle damage and inflammation

Our findings of elevated markers of muscle damage (CK and IL-6) following eccentric exercise support previous findings [32, 57]. Although variability in the response of muscle damage indicators (e.g. CK, IL-6) to eccentric exercise still persists [32], the simultaneous elevations in subsequent pain experienced (DOMS ratings) by participants suggests that the two (CK and IL-6 with DOMS) are linked, as demonstrated by Byrne et al. [44]. Our findings certainly support this relationship, as both increases in mean perceived muscle soreness (VAS) (Fig. 2) and reductions in pressure pain threshold exhibited similar trends as was found with the serum CK_Log_ levels (Fig. 4). VAS and serum CK_Log_ both showed peak increases at 24 h post-DHR, whilst the pressure pain threshold reached peak decrement at the same time point. Ormsbee et al. [32] indicated a significant time effect for CK following a single-bout of DHR in male athletes after supplementation with NO-Shotgun®. Our findings are consistent with these. Additionally, mean serum IL-6 levels increased post DHR, peaking immediately post and remaining elevated up to 72 h post (Fig. 5), however these findings were not significant. The findings of this study agree with others examining the effects of eccentrically-induced CK response to supplementation with whey protein [5, 53], BCAAs [17, 49], leucine [20, 51], creatine [21], but not others showing a reduction in CK as a result of creatine [58] or BCAA supplementation [49].

The mean response of hsCRP to the DHR showed a slight increase, with a significant decrease occurring between 24 (15.73% of baseline) and 48 h post-DHR (-5.44% of baseline), following which hsCRP levels increased beyond baseline (37.10% of baseline) (Fig. 6). Interestingly, although not significantly so, hsCRP continued to decrease below baseline in the MIPS group, experiencing a peak decline at 72 h post-DHR (-38.89% of baseline, moderate ES), while the CON group remained above baseline at all time points following the DHR with a moderate effect size at 72 h post-DHR). Similar findings were noted by Tara et al. [59] following supplementation with whey protein (89.3 g protein, 1.1 g fat, and 3.6 g CHO per 100 g), in which the group supplementing with whey protein experienced greater decreases in CRP compared to those supplementing with soy protein (83.3 g protein, 4.2 g fat, <2.1 g CHO, 175 mg isoflavones per 100 g). Previous studies have noted attenuation of CRP levels in response to regular exercise [60, 61], while increases in CRP have been noted in response to acute exercise protocols. Increases in CRP immediately post- and 24 h post-marathon have been evident in endurance trained males and females, with CRP levels returning to baseline two to six days post-marathon [62]. Several studies have suggested that the increase in CRP values is stimulated by the increase in the pro-inflammatory cytokine IL-6, which stimulates hepatic CRP stimulation [63, 64]. However, Michigan et al. [63] have indicated that the type of exercise and training may result in varied effects on CRP. Our findings do not support the notion that an increase in IL-6 increases the hsCRP response to EIMD. As is evident in Figs. 5 and 6, IL-6 peaked immediately post-DHR and hsCRP peaked 72 h post-DHR. Although it would appear that the MIPS did attenuate hsCRP response to the DHR, however, not significantly so, in comparison to an isocaloric placebo. These findings should be examined further in future research.

### Limitations

Low statistical power because of the modest sample size in the present study (*n* = 8) played a role in limiting the significance of the statistical comparisons conducted. A post hoc power analysis revealed that on the basis of the mean, between-groups comparison effect sizes observed in the present study (d = 1.1), an n of approximately 9 participants in each group was needed to obtain statistical power at the recommended .80 level. The strict inclusion criteria as well as performance of the DHR during the correct phase of the menstrual cycle and supplementation with a “muscle boosting” supplement, made recruitment for participants difficult. Although hormone levels were not tested to establish the phase of the menstrual cycle, verbal verification was obtained from the participants, and all participants were tested as close to the mid-follicular phase (7-11 days after menses) of the menstrual cycle as possible. This may have influenced the results obtained, as not all participants would have had similar levels of estrogen. Additionally, due to the small sample size, it was difficult to control for the use of contraceptives (oral, transdermal patch, vaginal ring), which would have impacted on the hormone levels and, as such, the time of testing; however during the testing period, participants did not report using any form of contraceptives. Furthermore, the indirect markers of muscle damage used in the present study may have been restricted with regards to their sensitivity to identify specific responses to the DHR and the effects of MIPS. It has been suggested that voluntary eccentric muscle actions do not result in muscle damage and inflammation, but rather provide evidence for myofibrillar remodelling and adaptation. Therefore, the use of an unbiased method to determine the degree of muscle adaptation is an important methodological concern to consider for future research [65, 66]. Although the sample size was small, it provided useful information for the planning of future larger studies.

## Conclusion

The results of this study are in accordance with others reporting increases in perceived muscle soreness, CK and IL-6, and decreases in pressure pain thresholds, squat jump performance, and hamstring flexibility following strenuous DHR exercise. The use of a propriety blend MIPS did not have an effect on markers of muscle damage, perceived soreness or performance compared to an isocaloric placebo, however, it may have attenuated the inflammatory response at 72 h post-DHR. Although numerous studies have documented results in attenuation of markers of muscle damage, perceived soreness, and improved performance with supplementation, other studies using protein, BCAAs, creatine and leucine have reported similar findings to ours. A key difference between our study and those supplementing with MIPS is that this study used endurance-trained female athletes compared to that of trained or recreationally active males in similar studies. However, the responses experienced in this study are similar to those using male participants, possibly indicating that the perceived muscle soreness, muscle damage markers and recovery following a single-bout of DHR do not vary between well trained males and females in terms of the responses to muscle damaging exercise.

## References

[1] Sousa M, Teixeira VH, Soares J (2014). Dietary strategies to recover from exercise-induced muscle damage. Int J Food Sci Nutr.

[2] Torres R, Ribeiro F, Duarte JA, Cabri JMH (2012). Evidence of the physiotherapeutic interventions used currently after exercise-induced muscle damage: systematic review and meta-analysis. Phys Ther Sport.

[3] Conceição MS, Libardi CA, Nogueira FRD, Bonganha V, Gáspari AF, Chacon-Mikahil MPT (2012). Effects of eccentric exercise on systemic concentrations of pro- and anti-inflammatory cytokines and prostaglandin (E2): Comparison between young and postmenopausal women. Eur J Appl Physiol.

[4] Rahbek SK, Farup J, de Paoli F, Vissing K (2015). No differential effects of divergent isocaloric supplements on signaling for muscle protein turnover during recovery from muscle-damaging eccentric exercise. Amino Acids.

[5] Cooke MB, Rybalka E, Stathis CG, Cribb PJ, Hayes A (2010). Whey protein isolate attenuates strength decline after eccentrically-induced muscle damage in healthy individuals. J Int Soc Sports Nutr.

[6] Cheung K, Hume PA, Maxwell L (2003). Delayed onset muscle soreness: Treatment strategies and performance factors. Sports Med.

[7] Sayers SP, Hubal MJ (2008). Histological, chemical, and functional manifestations of muscle damage. Skeletal Muscle Damage and Repair.

[8] Malm C, Sjödin B, Sjöberg B, Lenkei R, Renström P, Lundberg IE (2004). Leukocytes, cytokines, growth factors and hormones in human skeletal muscle and blood after uphill or downhill running. J Physiol.

[9] Miller PC, Bailey SP, Barnes ME, Derr SJ, Hall EE (2004). The effects of protease supplementation on skeletal muscle function and DOMS following downhill running. J Sport Sci.

[10] Flores DF, Gentil P, Brown LE, Pinto RS, Carregaro RL, Bottaro M (2011). Dissociated time course of recovery between genders after resistance exercise. J Strength Cond Res.

[11] Connolly DAJ, Sayers SP, McHugh MP (2003). Treatment and prevention of delayed onset muscle soreness. J Strength Cond Res.

[12] Sotiriadou S, Kyparos A, Mougios V, Trontzos C, Sidiras G, Matziari C (2003). Estorgen effect on some enzymes in female rats after downhill running. Physiol Res.

[13] Feng X, Li G, Wand S (2004). Effects of estrogen on gastrocnemius muscle strain injury and regeneration in female rats. Acta Pharm Sin.

[14] Stupka N, Lowther S, Chorneyko K, Bourgeois JM, Hogben C, Tarnopolsky MA (2000). Gender differences in muscle inflammation after eccentric exercise. J Appl Physiol.

[15] Rinard J, Clarkson PM, Smith LL, Grossman M (2000). Response of males and females to high force eccentric exercise. J Sports Sci.

[16] Chen WC, Huang WC, Chiu CC, Chang YK, Huang CC (2014). Whey protein improves exercise performance and biochemical profiles in trained mice. Med Sci Sports Exerc.

[17] Jackman SR, Witard OC, Jeukendrup AE, Tipton KD (2010). Branched-chain amino acide ingestion can ameliorate soreness from eccentric exercise. Med Sci Sports Exerc.

[18] Nicastro H, Carnauba RA, Massunaga ND, da Fonseca ABB, Paschoal V, Naves A (2014). Are the BCAAS/Leucine Supplementation Effects on Exercise-Induced Muscle Damage Related Immunity Response or to HMβ?. J Nutr Health Food Sci.

[19] Dale MJ, Thomson RL, Coates AM, Howe PRC, Brown A, Buckley JD (2015). Protein hydrolysates and recovery of muscle damage following eccentric exercise. Funct Foods Health Dis.

[20] Kirby TJ, Triplett NT, Haines TL, Skinner JW, Fairbrother KR, McBride JM (2011). Effect of leucine supplementation on indices of muscle damage following drop jumps and resistance exercise. Amino Acids.

[21] Rawson ES, Gunn B, Clarkson PM (2001). The effects of creatine supplementation on exercise-induced muscle damage. J Strength Cond Res.

[22] Howatson G, McHugh MP, Hill JA, Brouner J, Jewell AP, van Someren KA et al. Influence of tart cherry juice on indices of recovery following marathon running. Scand J Med Sci Sports. 2010;20(6):843–52. 10.1111/j.1600-0838.2009.01005.x 19883392

[23] Kuehl KS, Perrier ET, Elliot DL, Chesnutt JC (2010). Efficacy of tart cherry juice in reducing muscle pain during running: a randomized controlled trial. J Int Soc Sports Nutr.

[24] McLeay Y, Barnes MJ, Mundel T, Hurst SM, Hurst RD, Stannard SR (2012). Effect of New Zealand Blueberry consumption on recovery from eccentric exercise-induced muscle damage. J Int Soc Sports Nutr.

[25] Bishop D (2010). Dietary supplements and team-sport performance. Sports Med.

[26] Karp JR, Johnston JD, Tecklenburg S, Mickleborough TD, Fly AD, Stager JM (2006). Chocolate milk as a post-exercise recovery aid. Int J Sport Nutr Exerc Metab.

[27] Ferguson-Stegall L, McCleave E, Ding Z, Doerner III PG, Liu Y, Wang B et al. Aerobic exercise training adaptations are increased by postexercise carbohydrate-protein supplementation. J Nutr Metab. 2011:1–12. 10.1155/2011/623182 PMC3136187 21773022

[28] White JP, Wilson JM, Austin KG, Greer BK, St John N, Panton LB. Effect of carbohydrate-protein supplement timing on acute exercise-induced muscle damage. J Int Soc Sports Nutr. 2008;5:5. doi: 10.1186/1550-2783-5-5. 10.1186/1550-2783-5-5 PMC2288590 18284676

[29] Wilson JM, Kim J, Lee S, et al. Acute and timing effects of beta-hydroxy-beta-methylbutyrate (HMB) on indirect markers of skeletal muscle damage. J Nutr Metab. 2009;6:6. doi: 10.1186/1743-7075-6-6. 10.1186/1743-7075-6-6 PMC2642830 19193206

[30] Koot RW, Amelink MA, Blankenstein MA, Bar PR (1991). Tamoxifen and estrogen both protect the rat muscle against physiological damage. J Steroid Biochem Mol Biol.

[31] Clarkson PM, Hubal MJ (2002). Exercise-induced muscle damage in humans. Am J Phys Med Rehabil.

[32] Ormsbee MJ, Ward EG, Bach CW, Arciero PJ, McKune AJ, Panton LB. The Impact Of A Pre-Loaded Multi-Ingredient Performance Supplement On Muscle Soreness And Performance Following Downhill Running. J Int Soc Sports Nutr. 2015;12(2). doi: 10.1186/s12970-014-0063-6. 10.1186/s12970-014-0063-6 PMC4308018 25628519

[33] Shelmadine B, Cooke MB, Buford T, Hudson G, Redd L, Leutholtz B et al. Effects of 28 days of resistance exercise and consuming a commercially available pre-workout supplement, NO-Shotgun (R), on body composition, muscle strength and mass, markers of satellite cell activation, and clinical safety markers in males. J Int Soc Sports Nutr. 2009;6:16. doi: 10.1186/1550-2783-6-16. 10.1186/1550-2783-6-16 PMC2731073 19656392

[34] Ormsbee MJ, Mandler WK, Thomas DD, Ward EG, Kinsey AW, Simonavice E (2012). The effects of six weeks of supplementation with multi-ingredient performance suplements and resistance training on anabolic hormones, body composition, strength, and power in resistance-trained men. J Int Soc Sports Nutr.

[35] Cooke CB (2009). Kinanthropometry and Exercise Physiology Laboratory Manual: Tests, Procedures and Data.

[36] ACSM (2010). ACSM’s Guidelines for Exercise Testing and Perscription: Eighth Edition.

[37] Bobbert MF, Hollander AP, Huijing PA (1986). Factors in delayed onset muscle soreness of man. Med Sci Sports Exerc.

[38] Kinser AM, Sands WA, Stone MH (2009). Reliability and validity of a pressure algometer. J Strength Cond Res.

[39] Baltaci G, Un N, Tunay V, Besler A, Gerçeker S (2003). Comparison Of three different sit and reach tests for measurement of hamstring flexibility in female university students. Br J Sports Med.

[40] Casartelli N, Müller R, Maffiuletti NA (2010). Validity and reliability of the myotest accelerometric system for the assessment of vertical jump height. J Strength Cond Res.

[41] Starzak DE, Semple SJ, Smith L, McKune AJ. Differing cytokine responses by ethnic groups to a bout of exercise-induced muscle damage: a preliminary report. J Sports Med Phys Fit. 2016;56(6):665–77. 25692862

[42] Borg G. Borg's Perceived exertion and pain scales. Champaign: Human Kinetics; 1998.

[43] Dartnall TJ, Nordstrom MA, Semmler JG (2011). Adaptations in biceps brachii motor unit activity after repeated bouts of ccentric exercise in elbow flexor muscles. J Neurophysiol.

[44] Byrne C, Twist C, Eston R (2004). Neuromuscular function after exercise-induced muscle damage: Theoretical and applied implications. Sports Med.

[45] Eston RG, Mickleborough J, Baltzopoulos V (1995). Eccentric activation and muscle damage: Biomechanical and physiological considerations during downhill running. Br J Sports Med.

[46] Keane K, Salicki R, Goodall S, Thomas K, Howatson G. The muscle damage response in female collegiate athletes following repeated sprint activity. J Strength Cond Res. 2015. doi: 10.1519/JSC.0000000000000961. 10.1519/JSC.0000000000000961 25853920

[47] Etheridge T, Philip A, Watt PW (2008). A single protein meal increases recovery of muscle function following an acute eccentric exercise bout. Appl Physiol Nutr Metab.

[48] Greer BK, White JP, Arguello EM, Haymes EM (2011). Branched-chain amino acid supplementation lowers perceived exertion but does not affect performance in untrained males. J Strength Cond Res.

[49] Matsumoto K, Koba T, Hamada K, Sakurai M, Higuchi T, Miyata H (2009). Branched-chain amino acid supplementation attenuates muscle soreness, muscle damage and inflammation during an intensive training program. J Sports Med Phys Fit.

[50] Green MS, Corona BT, Doyle JA, Ingalls CP (2008). Carbohydrate-protein drinks do not enhance recovery from exercise-induced muscle injury. Int J Sport Nutr Exerc Metab.

[51] Stock MS, Young JC, Golding LA, Kruskall LJ, Tandy RD, Conway-Klaassen JM (2010). The effects of adding leucine to pre and postexercise carbohydrate beverages on acute muscle recovery from resistance training. J Strength Cond Res.

[52] McKinnon NB, Graham MT, Tiidus PM (2012). Effect of creatine supplementation on muscle damage and repair following eccentrically-induced damage to the elbow flexor muscles. J Sports Sci Med.

[53] Hoffmann JR, Ratamess NA, Tranchina CP, Rashti SL, Kang J, Faigenbaum AD (2010). Effect of proprietary protein supplement on recovery indices following resistance exercise in strength/power athletes. Amino Acids.

[54] Brown MA, Howatson G, Keane K, Stevenson E. Exercise-induced muscle damage following dance and sprint specific exercise in females. J Sports Med Phys Fit. 2016;56(11):1376–83. 26022746

[55] Bailey DM, Williams C, Betts JA, Thompson D, Hurst TL (2011). Oxidative stress, inflammation and recovery of muscle function after damaging exercise: effect of 6-week mixed antioxidant supplementation. Eur J Appl Physiol.

[56] Proske U, Morgan DL (2001). Muscle damage from eccentric exercise: mechanism, mechanical signs, adaptation and clinical applications. J Physiol.

[57] Gillum TL, Kuennen MR, Schneider S, Moseley P (2011). A review of sex differences in immune function after aerobic exercise. Exerc Immunol Rev.

[58] Cooper R, Naclerio F, Allgrove J, Jimenez A. Creatine supplementation with specific view to exercise/sports performance: an update. J Int Soc Sports Nutr. 2012;9(1):33. 10.1186/1550-2783-9-33 PMC3407788 22817979

[59] Tara MK, Park JS, Mathison BD, Kimble LL, Chew BP (2013). Whey protein but not soy protein, supplementation alleviates exercise-induced lipid peroxidation in female endurance athletes. Open Nutr J.

[60] Kasapis C, Thompson PD (2005). The effects of physical activity on serum C-reactive protein and inflammatory markers. J Am Coll Cardiol.

[61] Costa VB. Analysis of C-reactive protein levels after an eccentric exercise. Front Immunol Confer Abstr: 15th International Congress of Immunology. 2013. doi: 10.3389/conf.fimmu.2013.02.00040.

[62] Weight LM, Alexander D, Jacobs P (1991). Strenuous exercise: analogous to the acute-phase response?. Clin Sci.

[63] Michigan A, Johnson TV, Master VA (2011). Review of the relationship between C-reactive protein and exercise. Mol Diagn Ther.

[64] Córdova A (2010). Immunomodulators for inflammation and muscle injury due to exercise. Apunts Medicina de L’Esport.

[65] Malm C, Yu J-G (2012). Exercise-induced muscle damage and inflammation: re-evaluation by proteomics. Histochem Cell Biol.

[66] Yu J-G, Liu J-X, Carlsson L, Thornell L-E, Stål PS. Re-evaluation of sarcolemma injury and muscle swelling in human skeletal muscles after eccentric exercise. PLoS ONE. 2013;8(4):e62056. doi:10.1371/journal.pone.0062056. 10.1371/journal.pone.0062056 PMC3626686 23614012

